# Evaluation of musculoskeletal system injuries after the 2023 Kahramanmaraş Turkey earthquakes: Our single-center experience

**DOI:** 10.5339/qmj.2024.10

**Published:** 2024-02-21

**Authors:** Necmettin Turgut, Alaaddin Levent Özgözen, Salih Beyaz

**Affiliations:** 1 Başkent University, Adana Dr. Turgut Noyan Training and Research Center, Department of Orthopedics and Traumatology, Adana, Turkey Email: drnecmettinturgut@hotmail.com

**Keywords:** Earthquake, fracture, fasciotomy, amputation, crush syndrome

## Abstract

Introduction: This study aimed to retrospectively analyze patients who presented to the orthopedic and traumatology clinic following the 2023 Kahramanmaraş earthquakes.

Patients and Methods: Over a week after the earthquakes, two hundred and sixty patients were consulted at our clinic. Demographic data of the patients, duration of being under the rubble, fracture locations, types of surgeries performed, number of surgical sessions attended by individuals, and early mortality rate within one month were determined.

Results: The mean age of the patients was 40.2 ± 22.4 years. One hundred thirty-eight (53.1%) were female, and 122 (46.9%) were male. The average duration of being under the rubble was determined as 27.1 ± 28.0 hours. Sixteen patients died within one month after the earthquake. The one-month mortality rate among patients with orthopedic injuries was 6.15%. Forty-seven fasciotomies were performed in 35 patients, and 22 amputations were performed in 19 patients. The most injured region was the lower extremity (78 cases, 40%). The ratio of external and internal fixation in extremity fractures was 22%.

Conclusions: The management of musculoskeletal injuries can be successful with proper triage and treatment plans. Decisions regarding fasciotomy and amputation in patients with crush syndrome following an earthquake should be individualized. Implant sets should be planned accordingly, especially considering the higher occurrence of lower extremity injuries.

## Introduction

Earthquakes are one of the most devastating natural disasters, causing a significant impact on human lives, resulting in numerous deaths and injuries, and putting a strain on the healthcare system.^1^ Unlike other disasters, earthquakes are unpredictable and uncontrollable, making it difficult to take preventive measures in advance. More effective planning can be achieved in terms of both resources and personnel for future earthquakes by conducting epidemiological studies and analyzing the types of fractures, characteristics of injuries, treatments, and outcomes of patients admitted to hospitals and treated for earthquake-related injuries.

Turkey is in a primary earthquake zone, right in the heart of seismic fault lines, and has experienced significant earthquakes throughout its history. On 6 February 2023, two large-scale earthquakes, having a moment magnitude (Mw) of 7.7 at 04:17 am local time and of 7.6 at 13:24, centered in Kahramanmaraş, occurred, affecting approximately 14 million people in 11 cities in southern Turkey, and northern Syria. The first of these intense quakes lasted approximately 100 seconds, while the second endured for 45 seconds, collectively intensifying their destructive effects. According to the latest official data, there were at least 50,783 fatalities, over 122,000 injuries, and extensive damage. The triage, treatment, and management of the injured posed a challenging administrative task. Our hospital is located in Adana, within the earthquake zone. Compared to Maraş and Hatay, the most affected cities, Adana has been less affected, and healthcare services have largely continued without significant disruptions. Therefore, our hospital played a crucial role as one of the primary centers for post-earthquake healthcare services following the earthquake. Most of the patients coming to us were mainly from neighboring provinces, predominantly from Hatay. Factors such as the magnitude of the destruction, technical challenges, transportation difficulties, and prolonged rescue times from under rubble contributed to significant delays in patients reaching our clinic. Adana, Mersin, and Diyarbakir were selected as transport cities. After the initial care and surgeries, the patients were transferred to distant cities, not in the earthquake-affected area.

Following earthquakes, superficial injuries, fractures, open wounds due to high-energy trauma, compartment syndrome due to compression, crush injuries, and crush syndrome are frequently encountered, and patients are generally polytraumatized.^2^ It is crucial to apply prompt and effective treatment to earthquake victims because the survival rate is higher within the first 24 hours of receiving care, leading to reduced rates of complications. Early urgent fasciotomy is the gold standard treatment for acute compartment syndrome,^3,4^ the most encountered injuries, and some of the fasciotomies performed in this catastrophic environment may be incomplete. In delayed cases where early emergency fasciotomy could not be performed, treatments were individualized, leading to conservative follow-up for some patients. In contrast, others required delayed fasciotomies and decisions for amputation. Acute compartment syndrome can be defined as the disruption of circulation in a limited space due to increased pressure, impairing tissue functions and structures.^5^ Metabolites produced after crush injuries can lead to acute renal failure, which is frequently among the most common causes of death.

Post-earthquake first aid and triage organization are essential for the medical care of those rescued from under the rubble. After an earthquake, almost every type of orthopedic trauma can occur; therefore, triage should be prioritized in patients presenting to the emergency department. In such catastrophic situations, the shortage of personnel, time, and limited operating room and hospital beds necessitate this. As a result, we triaged patients with orthopedic injuries, quickly diagnosing their problems and prioritizing efforts to save lives and limbs. Planning all these surgeries and monitoring and evaluating patients require a multidisciplinary approach. In this study, we aimed to retrospectively describe and analyze the case series of patients presenting with musculoskeletal injuries following earthquakes and the subsequent treatment process.

## Materials and Methods

Two major earthquakes with magnitudes of 7.7 and 7.6 Mw, with their epicenters in the Pazarcik and Elbistan districts of Kahramanmaraş, occurred on 6 February 2023. Following the approval of the ethics committee of the Baskent University Institutional Review Board (KA23/195), the records of earthquake victims who were injured due to these earthquakes were examined at the Adana Dr. Turgut Noyan Research and Application Center, affiliated with Başkent University, between 6 February and 13 February. Patients who were consulted for their musculoskeletal traumas by the orthopedics and traumatology department and treated by our team were included in the study.

Patients were identified by searching consultation forms and the hospital information database system using the ICD-10 code X34 (earthquake victim). Those with incomplete medical information were excluded from the study. The patients’ demographic information (age, gender), time of admission, fracture localization and types, diagnosis codes, surgical notes, treatment methods, and mortality were evaluated. Mortality information and dates were obtained from the government’s death reporting system.

Serum creatinine kinase (CK) levels were assessed in all patients. Those with levels exceeding 1000 IU/l (normal values are 25–175 U/I) were considered in crush syndrome.

The patients or their relatives were contacted by phone to gather information about their general condition at the end of the three months after the quakes. The patients filled out three items on a 5-point Likert scale to assess a) their return to life, b) functional status, and c) pain levels.

## Results

Two hundred sixty patients were consulted in the orthopedics and traumatology department during the first week following the earthquake. The average age of the patients was 40.2 ± 22.4 years, with 138 (53.1%) identified as female and 122 (46.9%) as male. The average duration of being trapped under the rubble was determined as 27.1 ± 28.0 hours. There were 160 cases of crush syndrome, and 30 of them needed to undergo dialysis once or more. Within one month after the earthquake, 16 patients died. The one-month mortality rate for patients with orthopedic injuries was determined as 6.15%. It was determined as 9.38% for the 160 patients with crush syndrome. In 15 of these 16 patients, crush syndrome was present (3 with associated thoracic trauma, 2 with abdominal trauma, and 1 with abdominal and head trauma). When the causes of death in patients with crush syndrome were examined, they were observed as ARDS (n:3), sepsis (n:4), hyperkalemia (n:1), and acute renal failure (n:7). In the patient who died due to a non-crush-syndrome-related-cause, it was due to postoperative pulmonary embolism following intramedullary nailing surgery after hip fracture.

Fractures were classified by location, and a total of 194 separate fracture cases were observed (Table 1). The most common were spinal fractures (43 cases, 22.2%) and fractures of the cruris region (38 cases, 19.6%). Overall, when considering all lower extremity fractures (combining fractures in the femur, around the knee, cruris, and foot), they were observed more frequently in 78 cases (40%) compared to upper extremity fractures (n:55) (combining fractures in the around shoulder, humerus, around the elbow, forearm, and hand).

The number of surgery sessions performed on individuals were shown in (Table 2). The surgeries were classified according to their types (Table 3). Fasciotomies were performed in 47 cases on 35 patients, and amputations were performed in 22 cases on 19 patients. Of the fasciotomies, 31 were performed on the lower extremities (17 on the cruris, 8 on the thigh, 6 on the foot), and 16 were performed on the upper extremities (3 on the humerus, 10 on the forearm, 3 on the hand). Of the amputations, 16 were performed on the lower extremities (8 above the knee, 5 below the knee, 2 on the hip, 1 on the toe), and 6 were performed on the upper extremities (1 on the forearm, 2 on the elbow, 1 transhumeral, 1 on the shoulder, 1 on the finger).

For the remaining 118 patients who did not undergo surgical intervention, immobilization was applied in cases of fractures or significant soft tissue damage. In the presence of fractures, immobilization of long bone fractures, hand, and foot fractures was achieved using casts, while spinal fractures were treated with braces, and pelvic fractures required bed rest. Shoulder fractures were managed with sling treatments. Simple suturing and wound dressings for minor cuts or abrasions were performed in emergency outpatient clinics.

In patients undergoing primary amputation, the average length of hospital stay was determined to be 5.6 ± 3.5 days, while for fasciotomy patients, it was 16.1 ± 9.6 days. The mean Likert scale was significantly better in amputated patients than fasciotomy patients (3.6 and 2.7, respectively).

## Discussion

Earthquakes are among the leading disasters that cause orthopedic injuries. After an earthquake, patients with compartment syndrome, crush injuries, and crush syndrome pose the most challenging and urgent decisions in orthopedics. The lack of a clear consensus in the literature further complicates the situation for orthopedists. A multidisciplinary approach is necessary in disaster management after an earthquake, with orthopedists playing a central role. Following appropriate and systematic triage, successful outcomes can be achieved by employing suitable diagnostic and treatment methods. Therefore, life-saving measures should be prioritized, followed by limb-saving treatments and surgeries whenever possible. Success occurs through correct indication, proper timing, and appropriate practice.

Surgical treatment options such as fasciotomy and amputation, as well as the timing of these procedures, are controversial issues. Fasciotomy aims to reverse the damage to muscles and tissues, but as the duration after injury increases, the chances of success decrease, and secondary disadvantages such as infection and fluid loss due to fasciotomy also occur. Attempts to preserve the extremity in cases of infection and sepsis can increase vital risks. In cases where the time passed exceeds 48–72 hours, and vital risk becomes prominent, primary amputation is considered an alternative view, even suggesting that fasciotomy should never be performed. These opinions have been discussed in the literature.^6^ One of the most significant challenges in the decision-making mechanisms of orthopedists is the need to rely on subjective suspicion and experience due to the inability to perform intracompartmental pressure measurements used in diagnosing compartment syndrome during disasters such as earthquakes, tsunamis, and floods, where objective values for decision-making cannot be obtained. At the same time, the exact timing of compartment syndrome development and the time from injury when this condition occurs remain unknown. There are different views in the literature when comparing publications that favor or oppose fasciotomy, comparing patients who underwent fasciotomy with those who did not.^7,8^

The suggested time for fasciotomy after compartment syndrome is 8–10 hours, and the expected benefit decreases due to muscle necrosis after this period.^9^ After injury, a fasciotomy performed within the first 12 hours is termed “early,” while if performed later, it is referred to as “late” or “delayed” fasciotomy.^10,11^ However, in a disaster affecting large geographic areas and large population populations, such as the Maraş-Pazarcik earthquakes, unfortunately, a significant portion of the patients could only reach the hospital well after this critical 8–10 hour period. The collapse and damage of healthcare facilities in the cities most affected by the earthquake and the disruption of first aid services in this catastrophic environment delay the extraction of individuals from the rubble. Consequently, this has led to delayed fasciotomies and an increased tendency to change the decision in favor of amputation. The average entrapment duration for the patients in our study was 27.1 hours, which explains this situation. In a study conducted on the 2003 Bam earthquake, the entrapment duration was reported to be only 1.5 hours, explaining the completely different characteristics of the patients in that case series.^12^ Therefore, most fasciotomies in our study were late fasciotomies and were selected as personalized treatment methods for the patients. There is limited research on late fasciotomies, and there is a lack of information in the literature on this subject. When deciding on fasciotomy in a patient with a crush injury, the general condition of the patient, the presence of rhabdomyolysis, underlying diseases, and age should be taken into consideration, and a personalized approach should be adopted. However, it is believed that in cases where vital risk comes into play, in critical conditions, especially in cases where 48–72 hours have passed, it should not be forgotten that amputation can sometimes save lives. Social situations and medicolegal issues also pose problems in deciding for limb amputation and affect the decision to be made for the patient. It will be more accurate to decide on amputation with the joint decision of at least two orthopedists.^13^

In our clinic, we performed 22 primary amputations on 19 patients. We observed that they had a faster recovery, shorter hospitalization period, and an earlier return to normal life than fasciotomy patients. After examining the results of the three-item Likert scale three months after the earthquake, the group that underwent amputation was observed to have better outcomes.

Amputation and fasciotomy rates vary in the literature. Görmeli et al. applied 21 fasciotomies and 7 amputations in 46 crush injury patients in their series of 285 patients. In our case series, we performed 47 fasciotomies and 22 amputation surgeries on 260 patients.^14^ We attribute the increase in these numbers in our case series to our study’s higher number of crush syndrome patients (n: 160). Further scientific studies are needed to examine patients’ long-term outcomes and quality of life in this regard.

The timing of the earthquake is a significant determinant when examining the epidemiology of fractures occurring in earthquakes. Injuries mostly occurred when people were lying supine or in lateral decubitus positions in bed due to the first earthquake occurring at night. We believe that the fact that the lower extremity and spinal injuries were the most common injury locations in our case series is related to this situation. Previous studies have also identified the lower extremities as the most common injury location.^14,15^ Li et al., in their studies evaluating the 2016 Taiwan earthquake, reported that they encountered more thoracic cage and lower extremity injuries consistent with our case series in the earthquake that occurred at 03:57.^16^ Görmeli et al., in their article examining the 2011 Van earthquake, observed that the most common fractures were lower extremity fractures (46%). Consistent with the literature, lower extremity fractures were observed at a rate of 40.2% in our series.^14^ When planning materials for earthquakes, it should be considered that implant sets for femur, tibia, and spinal fractures, which are more frequently injured, are needed more. In our case series, the most performed surgical interventions were soft tissue surgeries (debridement, wound suturing, foreign body removal), consistent with the literature.^12^ Fasciotomy, amputation, arthroplasty, internal fixation, and external fixation are our other treatment methods. External fixation after an earthquake for damage control orthopedics offers a temporary successful stabilization method in open fractures, severe soft tissue injuries, and patients with crush injuries.^17^ In the literature, the use of external fixation for post-earthquake fractures has been reported at rates ranging from 2% to 30%.^18,19^ However, it should also be considered that this method leads to additional surgical procedures and prolonged hospital stays. We believe that the preferred stabilization method should be open reduction and internal fixation if the conditions of the hospital and the team are suitable when the patient’s soft tissue allows, and as a team, we planned our surgeries accordingly. The lower rate of external fixation compared to internal fixation in our series (%22) can be explained by our relatively good conditions: an adequate number of doctors and fluoroscopy devices and relatively lower soft tissue trauma in the patients who applied to us. However, each orthopedic team should evaluate the situation within their own conditions and prioritize external fixation if necessary.

There are some limitations in our study. Firstly, inherent limitations are related to the retrospective review of patient records due to the study’s retrospective nature. There is a lack of long-term patient outcomes, and accessing these data is challenging. As earthquakes are humanitarian disasters that force people to migrate, it has been difficult for us to follow up with the patients, and some of them migrated to different cities to continue their treatment after their initial treatment. Consequently, patients had to be contacted by phone. Another limitation is the relatively small number of patients in our study.

## Conclusions

In conclusion, the treatment of patients after earthquakes is hindered by the lack of treatment guidelines based on retrospective and observation-based studies with low scientific evidence. Treatment decisions should be personalized for each patient, and caution should be exercised in performing fasciotomy in patients who present late. The option of amputation as a life-saving measure should not be overlooked.

## Conflict of Interest

All authors declare that they do not have any conflict of interest either academic or financial.

## Figures and Tables

**Table 1.** The sites of the fractures.

Fracture location	Frequency (n)	Percentage (%)
**Upper extremity**	55	28.4
Around Shoulder	8	4.1
Humerus	17	8.8
Around elbow	4	2.1
Forearm	20	10.3
Hand	6	3.1
**Lower extremity**	78	40.2
Femur	26	13.4
Around knee	3	1.5
Cruris	38	19.6
Foot	11	5.7
**Spine**	43	22.2
**Pelvis**	18	9.3
**Total**	**194**	**100**

**Table 2.** The number of surgery sessions performed on individuals.

The number of sessions	N	Percentage(%)
**None**	118	45.4
**1**	95	36.5
**2**	27	10.4
**3**	9	3.5
**4**	4	1.5
**5**	5	1.9
**6**	2	0.8
**Total**	**260**	**100**

**Table 3.** Type of surgery.

Type of surgery	N	Percentage (%)
**Fasciotomy**	47	18.2
**Amputation**	22	8.5
**Soft tissue**	72	27.9
**External fixation**	15	5.8
**Internal fixation**	67	25.9
**Arthroplasty**	5	1.9
**Spine**	24	9.3
**Reduction**	6	2.3
**Total**	**258**	**100**
